# Integrating the relevance of sleep into testicular-cancer survivorship care

**DOI:** 10.1007/s00520-026-10697-9

**Published:** 2026-05-05

**Authors:** Mariana Toricelli, Tathiana A. Alvarenga, Allan Saj Porcacchia, Sergio Tufik, Monica L. Andersen

**Affiliations:** 1 Instituto Do Sono, Associação Fundo de Incentivo à Pesquisa, São Paulo, Brazil; 2 Departamento de Psicobiologia, Universidade Federal de São Paulo, São Paulo, Brazil

**Keywords:** Sleep, Cancer, Testosterone, Well-being

## Abstract

Sleep health, defined as a multidimensional construct encompassing regularity, duration, efficiency, satisfaction, timing, and daytime alertness, is a neglected pillar of testicular cancer survivorship. In parallel, common sleep disorders, including insomnia disorder and obstructive sleep apnea, remain underrecognized despite their relevance to cardiometabolic and endocrine health. This comment argues for the systematic integration of sleep screening and targeted interventions, such as CBT-I, into multi-disciplinary survivorship care. Recognizing sleep as a fundamental component of recovery offers a high-impact opportunity to optimize long-term clinical outcomes and quality of life for survivors.

Testicular cancer survivorship care has significantly evolved, prioritizing the management of long-term cardiometabolic and psychosocial toxicities. In this context, it is important to distinguish between sleep health and sleep disorders. Sleep health encompasses a multidimensional pattern of sleep and wakefulness, characterized by regularity, duration, efficiency, satisfaction, timing, quality, and daytime alertness, that supports physical and mental well-being. In contrast, sleep disorders, such as insomnia and obstructive sleep apnea (OSA), are clinical conditions that require specific screening, evaluation, and referral. Despite this distinction, both domains remain insufficiently integrated into current survivorship frameworks. For instance, recently proposed frameworks for testicular cancer follow-up continue to overlook sleep metrics, focusing predominantly on diet and physical activity [1]. The authors’ emphasis on fatigue, cardiorespiratory fitness, metabolic/cardiovascular risk, and mental health is timely. Yet a critical survivorship domain is missing from the trials summarized and the discussion: sleep. Humans sleep during 1/3 of a lifetime on average, representing an essential homeostatic process. Despite its close physiological and clinical coupling with fatigue, mood, endocrine recovery, inflammation, and cardiometabolic health, sleep was neither measured nor problematized across the included interventions. This omission constrains our mechanistic understanding of how exercise and psychological programs exert benefit in testicular cancer survivorship.

Drawing from evidence in broader oncology populations, both impaired sleep health and clinically significant sleep disorders are common and carry substantial consequences, but testicular cancer-specific survivorship data remain comparatively scarce. At the population level, disruptions across multiple dimensions of sleep health, including poor sleep quality or satisfaction, insufficient duration, and fragmented or irregular sleep, have been linked to fatigue, mood disturbances, inflammation, and adverse metabolic regulation. Clinically, sleep disorders warrant particular attention because they are prevalent, often underrecognized, and potentially modifiable contributors to symptom burden and cardiometabolic risk in survivorship care. A comprehensive meta-analysis including 160 studies (*n*≈46,000) estimated a pooled prevalence of clinically relevant sleep-related complaints and disturbances of approximately 61% in patients with cancer, underscoring a symptomatic burden that rivals pain and fatigue, and persists across the care continuum [2]. Classic syntheses similarly reported that 30-75% of newly diagnosed or recently treated patients endorse clinically relevant sleep complaints, at rates roughly double those in the general population [3]. More recent cohort-based work in survivors corroborates the persistence of disturbed sleep and its interdigitation with affective symptomatology and adverse outcomes [4, 5]. Insufficient or fragmented sleep amplifies sympathetic tone and hypothalamic-pituitary-adrenal axis activation, elevates pro-inflammatory cytokines, perturbs homeostasis of glucose and insulin, and suppresses anabolic hormonal signaling, pathways that directly intersect with the review’s focal outcomes [6]. Within this clinical domain, OSA, one of the most prevalent sleep disorders in men, represents a critical risk factor for cardiovascular disease and metabolic syndrome, thereby underpinning the aforementioned pathophysiological alterations [7], in addition to low testosterone levels and erectile complaints [8]. The characteristic intermittent hypoxia of OSA, along with inflammatory exacerbation and dysregulated metabolism, has been implicated in carcinogenic mechanisms in several types of cancer, potentially promoting tumor progression [9, 10]. Evidence directly linking OSA to testicular cancer biology remains limited. A large Korean claims-based study did not find a significant association between OSA and incident testicular cancer in the general adult male population overall, although an age-stratified signal was observed in men older than 65 years. Given the indirect nature of this evidence and the demographic mismatch with most testicular cancer survivors, we do not view this as survivorship specific evidence, but rather as hypothesis generating [11].

For testicular cancer survivors specifically, this physiology is not incidental: late hypogonadism and cardiometabolic vulnerability are well documented, and consistent human studies show that insufficient or fragmented sleep can depress androgenic signaling and aggravate metabolic risk, plausibly attenuating the benefits of exercise-based rehabilitation [12]. In healthy men, 1 week of sleep restriction lowered daytime testosterone by ~10-15% in a controlled protocol, and a meta-analysis indicates that ≥24h of total sleep deprivation reduces male testosterone; large observational and clinical datasets link poor sleep health, including reduced sleep quality, with hypogonadal symptoms and sexual dysfunction [13]. Mechanistically, sleep deprivation heightens sympathetic/hypothalamic-pituitary-adrenal-axis activity and inflammatory tone and worsens insulin sensitivity, pathways directly targeted by the exercise and cardiometabolic endpoints emphasized for this population. Although randomized rehabilitation trials in testicular cancer have not measured sleep, disease-specific survivorship cohorts report clinically relevant sleep complaints (e.g., “sleeping problems” in ~36% of long-term germ-cell tumor survivors), and older quality of life series already noted insomnia co-occurring with anxiety in nearly half of patients, underscoring that sleep is a material, not peripheral, determinant of survivorship health in this group [14]. A recent study in young adult testicular cancer survivors showed that cancer-related masculine threat was associated with poorer sleep quality, greater anxiety and depressive symptoms, and higher circulating IL-6 and CRP levels. It supports the view that sleep related complaints in this population are intertwined with psychological and biobehavioral burden rather than representing an isolated symptom [15].

The field stands to benefit from embedding sleep health within testicular cancer rehabilitation science. Future randomized trials should employ assessment tools explicitly aligned with the sleep-related vulnerabilities most plausible in this population. Given the coexistence of fatigue, psychological distress, endocrine disruption, and cardiometabolic risk among testicular cancer survivors, global sleep quality may be measured with the Pittsburgh Sleep Quality Index; insomnia symptoms with the Insomnia Severity Index; multidimensional sleep health with RU-SATED; overall sleep disturbance assessed by the PROMIS Sleep Disturbance scale; and day-to-day variability in sleep patterns with a sleep diary. Collectively, these instruments would allow investigators to determine whether the dominant signal in this population reflects poor overall sleep quality, insomnia-related symptoms, broader impairment in sleep health, or irregular sleep timing and routine instability.

Trials should also incorporate unobtrusive, objective assessment using actigraphy, with prespecified evaluation of sleep duration, efficiency, fragmentation indices, timing variability, midpoint, and regularity metrics, and, when circadian disruption is of interest, rest-activity rhythm measures. These variables are clinically meaningful because they can be linked to fatigue, endocrine dysregulation, and cardiometabolic vulnerability. In parallel, trials should include mediation analyses to examine whether improvements in these sleep dimensions partially transmit the effects of exercise and psychological interventions on fatigue and cardiometabolic risk. Investigators should systematically test dedicated sleep-targeted components within multimodal rehabilitation frameworks already advocated for this population. These components may include cognitive behavioral therapy for insomnia (CBT-I), circadian-aligned light timing, and structured wake-sleep scheduling (Fig. 1) [16].

**Fig. 1:**
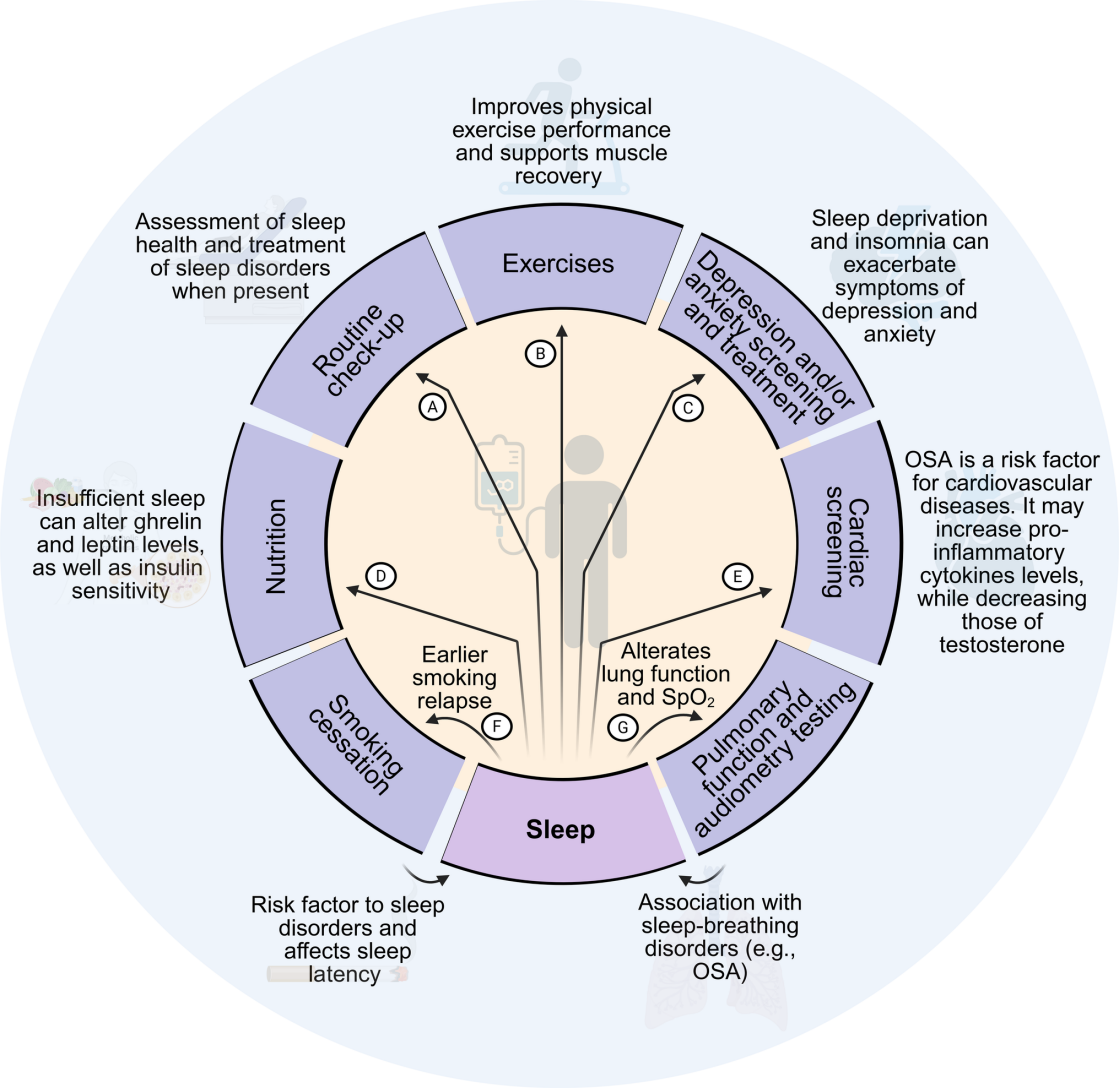
Relationship between sleep and prehabilitation/survivorship strategies in patients with testicular cancer. **A** Sleep health should be routinely assessed during survivorship visits through structured questions and validated tools matched to the intended construct, such as multidimensional sleep health, global sleep quality, insomnia symptoms, and day-to-day sleep patterns. Objective testing should be indication driven, with polysomnography reserved for survivors with suspected obstructive sleep apnea or other sleep disorders requiring formal diagnostic evaluation and referral. **B** Adequate sleep supports exercise capacity, recovery, and anabolic regulation, all of which are relevant to rehabilitation outcomes in testicular cancer survivorship. In contrast, insufficient or fragmented sleep may impair physical performance, delay recovery, and aggravate fatigue and pain. **C** Sleep complaints in testicular cancer survivors should not be viewed in isolation, as poor sleep health and sleep disorders frequently coexist with anxiety and depressive symptoms. Insomnia, circadian disruption, and unstable sleep patterns may intensify psychological distress, while anxiety and depression may further disrupt sleep, reinforcing a bidirectional cycle with implications for symptom burden and survivorship care. **D** Sleep and metabolic regulation are closely interconnected. Late and high caloric food intake may impair sleep quality and favor weight gain, whereas insufficient or irregular sleep may alter hypothalamic-pituitary-adrenal axis activity, leptin and ghrelin signaling, and insulin sensitivity, thereby increasing appetite for energy-dense foods and contributing to adiposity and cardiometabolic risk. **E** Obstructive sleep apnea deserves particular attention in survivorship care because it is associated with hypertension, endothelial dysfunction, systemic inflammation, and reduced testosterone levels. Conversely, cardiopulmonary conditions may worsen sleep initiation and maintenance, creating a reciprocal pathway through which sleep disorders and cardiovascular burden may amplify one another. **F** Smoking may contribute to poor sleep health by increasing sleep latency and by worsening insomnia symptoms and sleep-disordered breathing risk. Sleep disruption during tobacco withdrawal may complicate cessation efforts, making sleep assessment clinically relevant in survivors attempting to stop smoking. **G** Impaired pulmonary function may increase vulnerability to sleep-related breathing disorders, while sleep-disordered breathing may in turn worsen nocturnal oxygenation and aggravate respiratory symptoms. This bidirectional relationship is relevant when respiratory limitation coexists with fatigue, reduced exercise tolerance, or suspected sleep apnea. Abbreviations: OSA = obstructive sleep apnea, SpO_2_ = peripheral capillary oxygen saturation. Elaborated with BioRender

Initial studies in this population may need to prioritize feasibility and implementation. These efforts should test whether sleep assessment and targeted interventions can be integrated into routine survivorship care before large-scale efficacy trials are undertaken. Pragmatic comparators could include usual survivorship care, attention-matched control, or stepped-care approaches, with candidate endpoints spanning sleep outcomes, fatigue, mood, endocrine measures, and cardiometabolic markers. At present, survivors reporting sleep problems are rarely managed through clearly defined sleep-specific pathways within testicular cancer follow-up. In routine survivorship care, polysomnography would not often be a first-line assessment tool. Instead, it represents a referral-based diagnostic step for survivors with symptoms suggestive of OSA or other clinically significant sleep disorders.

Incorporating these elements would impose minimal burden while offering mechanistic insight and potential clinical impact. Testicular cancer survivorship pathways currently monitor endocrine, metabolic, cardiovascular, and psychosocial sequelae without explicitly embedding sleep assessment into regular follow-up. From a practice perspective, this remains a key gap, considering that sleep occupies roughly 1/3 of the daily period. A more comprehensive model would pair multidimensional sleep-health assessment with targeted screening and referral pathways for insomnia disorder and OSA, in parallel with monitoring of testosterone, lipids, glucose, blood pressure, fatigue, and mental health.

Briggs et al. have advanced the rehabilitation agenda for testicular cancer survivors. We respectfully suggest that explicitly integrating sleep into this framework will sharpen mechanistic inference, enhance symptom relief and quality of life, and potentially magnify the durability of benefits attributed to exercise and psychological support. In a population facing decades of survivorship, overlooking sleep risks leaving meaningful health gains unrealized.

## Data Availability

Data sharing not applicable to this article as no datasets were generated or analyzed during the current study.
